# Influence of connective tissue grafts on implants in the aesthetic area: A systematic review. Are connective grafts essential?

**DOI:** 10.4317/jced.61668

**Published:** 2024-06-01

**Authors:** Rocío Durante-Lacambra, Ricardo-Bahram Taheri, María-Isabel Leco-Berrocal, Carmen López-Carriches

**Affiliations:** 1 Doctor of Dental Surgery. DDS. Collaborator. School of Dentistry. Universidad Complutense de Madrid. Spain; 2 Assistant Professor. Department of Dental Clinic Specialties. School of Dentistry. Universidad Complutense de Madrid. Spain; 3 Associate Professor. Department of Dental Clinic Specialties. School of Dentistry. Universidad Complutense de Madrid. Spain

## Abstract

**Background:**

Achieving adequate aesthetics with implants in the anterior sector continues to be a challenge. One of the most studied and currently used techniques is the use of autologous connective tissue grafts to improve the peri-implant soft tissues in this area. Our objective is to analyze whether these techniques have a predictable impact on the tissues and aesthetics; and if it is worth performing them.

**Material and Methods:**

A bibliographic search was carried out, including different digital portals.

**Results:**

A total of 8 articles were analyzed. This procedure did not have an impact on the hard tissue but did have an impact on gingival recessions and soft tissue thickness. Regarding aesthetics, the results are controversial. It seems that they can slightly improve the PES (Pink Esthetic Score or Pink Index). Also, a negative impact on the texture of the soft tissue has been found.

**Conclusions:**

It is necessary to individualize each case (especially depending on the gingival biotype) since taking a connective tissue graft from the palate entails discomfort for the patient, and this technique is not free of complications.

** Key words:**Dental implants, aesthetics, connective graft.

## Introduction

Replacing a tooth in the anterior sector of the maxilla with implants is a great challenge. In recent years, numerous techniques have emerged aiming to increase peri-implant keratinized soft tissues to improve aesthetics. However, there is still a lack of scientific evidence to quantify the impact these procedures have and justify their use. These techniques entail morbidity in the patient, postoperative discomfort, increased surgical time, and extra cost.

The main objective of this study is to revise the current scientific evidence to justify the use of autologous connective tissue grafts to achieve esthetics in the anterior maxilla.

## Material and Methods

A bibliographic search was performed between two reviewers (RD, CL) in parallel, using different databases (PubMed, Cochrane, Scopus and Google Scholar), including publications in English until the 1st of March 2023. The MESH terms used were: “dental implants, aesthetics, connective tissue graft, soft tissue graft , free gingival graft , gingival autograft ” These terms were searched with the Boolean operators “AND” and “OR”. The PRISMA guide was followed to answer the PICO question (1) and the statistics used were descriptive due to the characteristics of the study.

The research question was: “Is the use of connective tissue grafts effective to improve aesthetics on implants placed on the anterior sector of the maxilla? The abbreviations PICO was followed: P (population) = patients over 18 years of age treated with implants in the maxillary aesthetic sector; I (intervention) = surgical intervention for implant placement with connective tissue grafts; C (comparison) = comparison of hard tissue (bone), soft tissue, and esthetic gain or loss with respect to subjects not treated with grafts; O (outcomes)= marginal bone level (MBL), mid-buccal gingival level (MBGL), buccal peri-implant soft tissue thickness (BTT), Gingival recession (GR) and aesthetic result with PES (pink esthetic score); S ( study design ) = randomized controlled trials.

The inclusion criteria were: Randomized clinical studies in humans with more than four cases, implants located in the anterior sector of the maxilla, results with numerical data referring to marginal bone level and/or gum thickness and/or gingival recession and/or pink esthetic score, connective tissue grafts and control group without graft or with xenograft, minimum follow-up of 12 months.

The exclusion criteria: Patients with smoking habits, systemic diseases or diabetics, implants without a minimum follow-up of twelve months, *in vitro* studies, studies that did not include data for bone or soft tissue gain, loss, or pink esthetic score.

To select the articles, the following parameters were independently analyzed: year of publication, type of study, follow-up time, number of patients, number of implants, implant position in the arch, immediate/delayed implant, MBL, GR, soft tissue thickness and PES.

Results and Discussion

**Figure 1:**
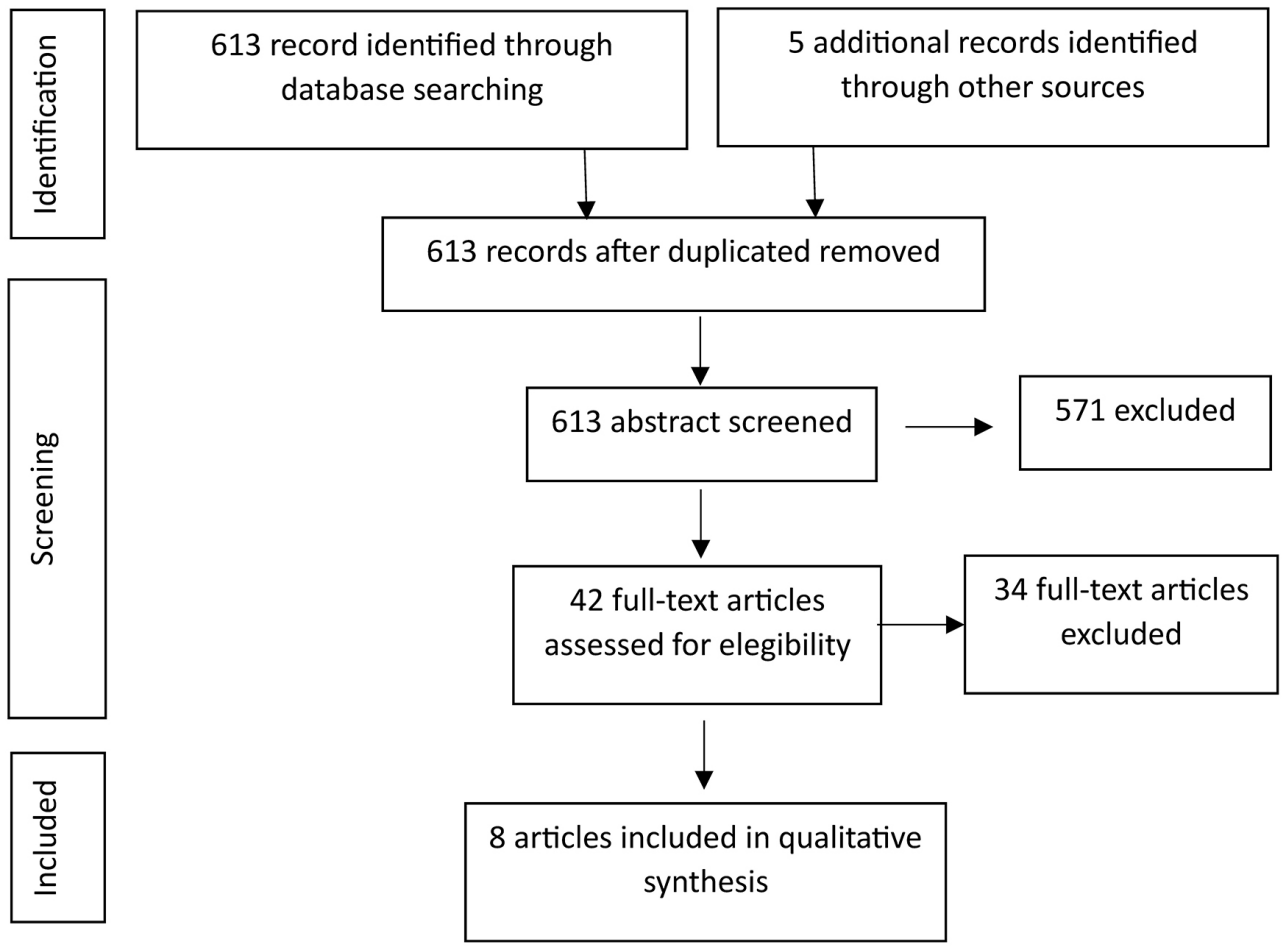
Prisma flow diagram of included articles.

A total of 613 studies were identified in the initial search. Other 5 articles were identified via manual search. 5 duplicate articles were removed. Two authors (RD and CL) reviewed all the abstracts and selected those related to the research question. In this phase, 571 articles were discarded. After excluding by abstract, 42 articles were read in full text. Only 8 articles met the inclusion criteria, (Fig. 1).  Table 1 summarizes the selected articles.

Marginal bone level was measured in five studies, in a total of 248 patients. No significant differences were found in any of them. However, there was a decrease of MBL in the graft free study groups, ( Table 2).

The gingival recession and mid-buccal gingival level were measured in five studies, including a total of 260 patients. Wouter and all (9), found a significant improvement of the mid-buccal gingival level in the graft group (0.2 ± 0.7mm) compared to the control group (-0.48 ± 1.13mm) (*p*=0.014).

In addition, they found greater recessions (32%) in the group without graft than in the control group (8%). On the other hand, in the study by Yoshino (8) it was found that the change in the gum was greater in the graft free group, ( Table 3).

Regarding buccal peri-implant soft tissue thickness, significant differences were found in the study by Abdelwahab (3). There was a thickness of 1.5 ± 0.4 mm in the grafted implants, compared to 0.4 ± 0.2 mm in the control group (*p*=<0.001). Additionally, in the study by Frizzera (4), a greater thickness was achieved when the graft was performed, however these changes were not significant. The Pink Esthetic Score variable was measured in six of the selected studies, with a total of 274 patients. Regarding the total value, significant differences were only found in the study by Migliorati (2), where the aesthetic result was greater in the group that had received connective tissue grafts. These values were even higher in the case of fine biotypes, where aesthetics was worse in both groups.

On the other hand, in the rest of the studies no differences were found. Only the soft tissue texture score was higher in the control group. The same finding was seen by Zuiderveld (5). In the study by Frizzera (4), higher PES values were found in the study group (although not significant); with smaller recessions. Wouter (9) found no differences in the total PES. They did find a worse score for gingival margin esthetics and texture in the group with graft, ( Table 4).

Connective tissue grafts can indeed be effective in improving aesthetics in the anterior sector; but they are not necessary in all cases. When planning, the periodontal biotype and the thickness of the tissues must be considered before performing the graft. It is known that thin biotypes are the most prone to recession and worse aesthetic results, which is why they are candidates to these techniques.

In addition, it must be considered that there are other less invasive methods (such as the use of immediate provisional) that also make it possible to reshape the peri-implant tissues to improve aesthetics (10). This technique eliminates the need to extract an autologous graft from the palate and therefore decreases the mobility. Other alternatives techniques include the use of collagen matrices or porcine acellular dermal matrices (11). Although evidence is still limited to confirm their effectiveness to replace connective tissue grafts. It must also be considered that the long-term stability of the graft is still not entirely clear (3), so further long-term follow-up studies are needed.

We can conclude that the use of connective tissue grafts has little impact in the bone level, although it may contribute to less bone loss after implant placement.

Connective tissue grafts improve gingival recessions and reduce the incidence of recessions.

Gingival thickness increases after graft techniques. More research is needed to demonstrate this quantitatively and to study whether this thickness is maintained in the long term.

The impact on aesthetics is not yet evident, in most studies the differences are not significant. They can improve the aesthetics of the margin, but they usually worsen the texture.

The gingival biotype plays a key role in the result, and it is necessary to take it into account in the design of the experimental studies.

## Figures and Tables

**Table 1:** Description of the selected articles.

Author/ year of publication	Follow-up (months)	Nº Patients	Age of patients in years	Test group	Control group	Number of implants	Placement of implants	Gingival biotype	Inmediate/Delayed load of implant
Migliorati (2015) (2)	12	48	47,5	Connective tissue graft	Inmediate implant placement (no graft)	48	Central Incisors	Test: 14 thin; 10 thick No Graft: 12 thin; 11 thick	Inmediate implant placement/ with provisional
Abdelwahab (2022) (3)	12	21	43,4	Connective tissue graft	Inmediate implant placement (no graft)	22	Incisors (4)Canines (4)Premolars (14)	Not reported	Delayed implant placement/ with provisional
Frizzera (2019) (4)	12	24	23-65	Connective tissue graft Collagen matrix	Inmediate implant placement (no graft)	24	Central incisors	Test: 5 thin; 3 thick Collagen matrix: 4 thin; 4 thickControl: 5 thin; 3 thick	Inmediate implant placement/ with provisional
Zuiderveld (2018) (5)	12	58	45,5	Connective tissue graft	Inmediate implant placement (no graft)	60	Incisisors (47)Canines (10)Premolars (3)	Test: 20 thin; 10 thick Control: 15 thin; 15 thick	Inmediate implant placement/ with provisional
Zuiderveld (2018) (6)	12	60	47,5	Connective tissue graft Collagen matrix	Inmediate implant placement (no graft)	60	Incisors (51)Canines (4)Premolars (2)	Test: 13 thin; 7 thick Collagen matrix: 10 thin; 10 thickControl: 15 thin; 5 thick	Inmediate implant placement/ with provisional
Zuiderveld (2020)(7)	12	55	46,2	Connective tissue graft	Inmediate implant placement (no graft)	60	Incisors (44)Canines (8)Premolars (3)	Test: 18 thin; 10 thickControl 13 thin; 14 thick	Inmediate implant placement/ with provisional
Yoshino (2014) (8)	12	20	52,6	Connective tissue graft	Inmediate implant placement (no graft)	20	Incisors (13)Canines (3)Premolars (2)	Test: 0 Thin; 10 thick Control: 3 thin; 7 thick	Inmediate implant placement/ with provisional
Wouter (2018) (9)	12	60	46,6	Connective tissue graft	Inmediate implant placement (no graft)	60	Incisors (28)Canines (10)Premolars (3)	Test: 20 thin; 10 thickControl: 15 thin; 15 thick	Inmediate implant placement/ with provisional

**Table 2:** Marginal bone level results. MBL = marginal bone loss.

Author/ year of publication	Test (MBL, marginal bone loss mm )		Control (MBL, marginal bone loss mm )	
	Mean	SD	Mean	SD
Migliorati (2013)(2)	0,001	0,092	-0,136	0,107
Abdelwahab (2022)(3)	Not reported		Not reported	
Frizzera (2019)(4)	Not reported		Not reported	
Zuiderveld (2018)(5)	Mesial: 0,9 / Distal: 0,8	Mesial: 0,4 - 1,2 / Distal: 0,0 -1,1	Mesial: 0,8 / Distal: 0,8	Mesial:0,5- 1,2 / Distal: 0,0-1,1
Zuiderveld (2018) (6)	Mesial: 0,3 / Distal: 0,5	Mesial: 0,0-0,9 / Distal: 0,0-1,0	Mesial: 0,5 / Distal: 0,4	Mesial:0,0-0,9 / Distal: 0,0-1,0)
Zuiderveld (2020)(7)	0,05	± 0,33	0,01	0,38
Yoshino (2014)(8)	–0.01	± 0.27 mm	–0.14	± 0.53 mm
Wouter (2018)(9)	Not reported		Not reported	

**Table 3:** Gingival recession and Mid-buccal gingival level.

Author/ year of publication	Test (Gingival recession and mid-buccal gingival level)		Control (Gingival recession and mid-buccal gingival level)	
	Mean	SD	Mean	SD
Migliorati (2015)(2)	Not reported		Not reported	
Abdelwahab (2022)(3)	Not reported		Not reported	
Frizzera (2019)(4)	Not reported		Not reported	
Zuiderveld (2018)(5)	0,1	±0,8	-0,5	±1
Zuiderveld (2018)(6)	-0,03	±0,2	0,15	±0,2
Zuiderveld (2020)(7)	0,07	0,33	-0,52	±1,96
Yoshino (2014)(8)	-0.25	± 0.35	–0.7	± 0.48
Wouter (2018)(9)	0,2	0,7	-0,48	±1,13

**Table 4:** Buccal peri -implant soft tissue thickness and Results PES= pink aesthetic score or pink aesthetic.

Author/ year of publication	Test (Buccal peri -implant soft tissue thickness)		Control (Buccal peri -implant soft tissue thickness)		Test PES		Control PES	
	Mean	SD	Mean	SD	Mean	SD	Mean	SD
Migliorati (2015)(2)	1,8	0.8	1.1	0,5	Not reported		7,15	
Abdelwahab (2022)(3)	1,5	0,4	0,4	0,2	10,7	1	10,6	1,6
Frizzera (2019)(4)	3,04	0,61	2,11	0,6	10,75	1,38	9,87	1,64
Zuiderveld (2018)(5)	Not reported		Not reported		6,4	1,5	6,8	1,5
Zuiderveld (2018) (6)	Not reported		Not reported		6,6	1,5	7	2,4
Zuiderveld (2020)(7)	Not reported		Not reported		Not reported		Not reported	
Yoshino (2014)(8)	Not reported		Not reported		Not reported		Not reported	
Wouter (2018)(9)	Not reported		Not reported		11,28	1,67	11,36	1,65

## Data Availability

The datasets used and/or analyzed during the current study are available from the corresponding author.
